# Distal Radial Fracture Fixation in Adults using Intramedullary Elastic Wires Augmented with either Cast Immobilisation or External Fixation

**DOI:** 10.5704/MOJ.2111.006

**Published:** 2021-11

**Authors:** P Gadegone, W Gadegone, V Lokhande, N Jawrani

**Affiliations:** 1 Department of Orthopaedics, Lokmanya Tilak Municipal General Hospital and Lokmanya Tilak Municipal Medical College, Mumbai, India; 2 Department of Orthopaedics, Government Medical College, Chandrapur, India; 3 Department of Orthopaedics, Smt. Kashibai Navale Medical College and General Hospital, Pune, India; 4 Research and Development, Jawrani MedTECH Consulting, Mumbai, India

**Keywords:** distal radial fracture, closed reduction, intramedullary, antegrade elastic wires, external fixation

## Abstract

**Introduction:**

The aim of this study was to evaluate the clinical outcomes following treatment of distal radial fractures using intramedullary elastic wires with a combination of either cast immobilisation or external fixation.

**Materials and methods:**

A total of 42 patients (24 females and 18 males) aged 40 to 78 years who presented with displaced and unstable, closed or grade I open, extra- and/or intra-articular fractures of the distal radius were included in the study. Twenty-seven fractures were AO/OTA Type A2-A3 and 15 Type C1-C2. Twenty-four patients were treated with antegrade intramedullary (IM) fixation with elastic wires followed by cast immobilisation and 18 required an external fixator in lieu of casting.

**Results:**

Final follow-up evaluation was conducted 12 months post-surgery using Sarmiento's modification of Lindstrom criteria and the demerit point system of Gartland and Werley. Successful fracture union was observed in all patients between eight to 14 weeks. Using Sarmiento's modification of Lindstrom criteria, 12 patients (28.6%) had excellent, 23 (54.8%) had good and 7 (16.6 %) had fair results. Based on the functional evaluation using the demerit point system of Gartland and Werley, 13 patients (31%) had excellent, 25 (59.5%) had good and four (9.5%) had fair results. None of the patients had a poor outcome using either of these criteria. Although a fracture union rate of 100% was confirmed clinically and radiographically, eight out of the 42 patients had minor complications in our study. One patient had uneventful IM migration of the wires, one patient reported a feeling of wire loosening, three patients complained of joint stiffness and soft tissue irritation, and three others reported on-going pain. The total cost of all implants used per case was less than INR 1,000.

**Conclusions:**

Good to excellent functional and radiographic outcomes with easy to manage complications are achieved with the techniques described. Patient selection is key to determining which particular method should be prescribed in a given case.

## Introduction

Fractures of the distal radius are a common occurrence in both the young and elderly and the majority are treated with casting alone. In the case of a closed reduction followed by cast immobilisation, loss of reduction and stability can occur without being noticed until the cast is removed. If the fracture has united in a mal-aligned or un-desired orientation, it can result in poor clinical outcomes in the long-term^1^. Nonetheless, closed reduction is still the primary choice of treatment for most surgeons since it is minimally invasive, cost-effective and continues to result in good clinical outcomes.

Once non-surgical treatment options are exhausted, or determined to be inadequate for a given case, surgery is the only option, and a multitude of methods are available at the disposal of the treating orthopaedic surgeon^2-4^. In the case of a distal radial fracture fixation, any surgery must restore radial length, alignment and inclination, and ensure that the distal radial articulating surface is adequately and accurately reduced, and that desirable range of motion is achieved^5^. The use of bone plates and screws has been gaining popularity in the last few decades but has its own advantages and disadvantages^6,7^. Minimally invasive treatments that are cost-effective are preferred by many surgeons for their economically under-privileged patients^8^. The percutaneous pinning technique with K-wires is one such option for treating extra-articular, with or without simple intra-articular, fractures of the distal radius^9^. However, soft tissue irritation, algodystrophy, pin tract infection, injury to radial sensory nerves and extensor tendons with this technique continue to be reported in the literature^10,11^. Another option for such fractures is an intramedullary (IM) nail with supplementary screw fixation. This minimally invasive implant can result in reduced implant irritation, lower post-operative pain, and provides a strong and stable fixation enabling early range of motion exercises at the wrist joint. However, these too are fraught with complications such as injury to the superficial branch of the radial nerve, screw penetration into the distal radio-ulnar joint, and potential loss of reduction^12^.

A novel, yet cost-effective technique as described by Sato *et al* involves antegrade IM placement of wires for fixation of distal radial fractures^13^. These wires exhibit a certain degree of elasticity. This technique has shown promise as it results in good to excellent clinical outcomes while minimising some of the complications noted with the other aforementioned techniques^14^. Thus, the aim of our study was to evaluate the clinical safety and effectiveness of closed fracture reduction technique followed by fixation using antegrade elastic IM wires with or without augmentation with an external fixation apparatus used for treating closed or Grade I (Gustilo-Anderson Classification) extra-articular and simple intra-articular fractures of the distal radius. In cases in which an external fixator was not used, cast immobilisation was applied. We hypothesise that patients would achieve satisfactory clinical outcomes with a manageable set of complications following either of these two treatment methods.

## Materials and Methods

In compliance with our hospital’s standard practices, approval for this prospective study was sought from the human research ethics committee. The inclusion criteria were adult patients presenting with displaced and unstable closed or Grade I open (Gustilo-Anderson Classification) fractures within two weeks post-injury and having a high probability of being successful candidates for a closed reduction technique augmented with minimal surgical fixation. The exclusion criteria were patients presenting with grades II-III open, externally irreducible, displaced, severely comminuted and intra-articular fractures, and patients presenting with pre-existing impairment of the involved hand and wrist function, mental or physical inability to cooperate with post-operative rehabilitation protocol, bilateral fractures, polytrauma and having had previous fracture(s) in the same limb. A written informed consent for participation in the study was obtained from all 42 patients who met these criteria, and was conducted between December 2015 to December 2019. The mean age of patients at the time of surgery was 63 years (range: 40 to 78 years). Twenty-four patients were females and 18 males. All 42 patients presented with a fracture of the distal radius. Twenty-two had sustained injury due to domestic fall, 18 due to road traffic accident and two were job related. There were 27 cases of AO/OTA Type A2-A3 fractures and 15 cases of Type C1-C2. The average injury to surgery duration was 2.6 days (range: 1 to 9 days). Average operating time was 45 minutes, and the average hospital stay was two days. Table I summarises patient demographics and fracture classification data.

**Table I:** Patient demographics and fracture classification

Number of Patients	42
Male	24
Female	18
Average Age (range) @ time of surgery	63 years (range: 40 to 78 years)
Number of Patients with Fracture in the Dominant Hand	34
AO/OTA Fracture Classification	
A2	18
A3	9
C1	12
C2	3

**Fig. 1:**
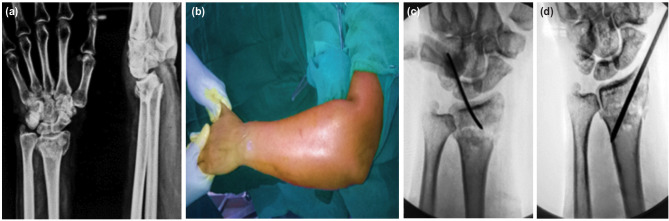
(a) Images showing unstable extra-articular distal radial fracture in both AP and lateral views, (b) closed reduction being attempted with counter-traction, (c) intra-articular K-wire for maintaining the reduction, (d) additional temporary K-wire for stabilisation of the reduced fracture until final fixation.

In all cases, the surgery was performed under a brachial block anaesthesia. The patient was positioned supine with the shoulder abducted to 90°, elbow flexed to 90°, forearm pronated and wrist in neutral position. After routine skin preparation and draping, closed reduction of the fracture was carried out. In some of the more challenging cases, a 2.5mm K-wire was introduced percutaneously from the styloid process, across the fracture and into the opposing radial cortex (Fig. 1) to achieve satisfactory fracture stabilisation. In all cases, the quality and acceptable criteria of reduction, were fluoroscopically confirmed.

**Fig. 2:**
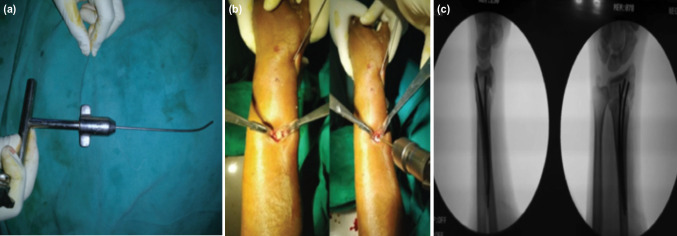
(a) Images showing a pre-bent wire prior to insertion, (b) exposure and drilling of radius for antegrade placement of wires, (c) and AP and lateral radiographs after final placement of the wires.

Next, surgical insertion of the stainless steel elastic wires was performed. A 3cm longitudinal incision was made 8cm to 10cm proximal to the radial styloid over the dorsal-radial aspect of the radius bone. The space between extensor carpi radialis brevis muscle and the extensor digitorum muscle proximal to the abductor pollicis longus muscle was developed to access the cortex of the radius dorsal to the pronator muscle. Depending on the dimension of the radius in a given patient, either a 2mm or a 2.5mm drill was used to create two holes at two different sites, radial and ulnar, in the radius. After starting the drill perpendicular to the bone to gain access through the cortical shell, it was directed obliquely at an angle of approximately 40° to 45° as recommended by Mostafa^14^. Care was taken to avoid penetration of the opposite cortex. Next, three 2mm pre-bent stainless steel elastic wires with blunt tips were manually inserted through these holes into the IM canal with the aid of a T-handle drill. The wires were directed distally until they were placed across the fracture site towards styloid process in the metaphyseal bone while the closed reduction was maintained. As the wires were being inserted through the IM canal, they formed a smooth curve due to their elastic properties, thus conforming to the shape of the radial canal. The wire placed at the ulnar aspect was rotated 180° and directed towards the ulnar side of the distal radial fracture fragment. Care was taken to ensure that the fractured segment at the mid-articular level was supported by at least one wire. All wires were advanced only till the level of the subchondral bone, sufficient to ensure that the fractures fragments were “captured” by the wires. The proximal ends of the wires were then bent and cut near the bone prior to closure of the incision (Fig. 2).

**Fig. 3:**
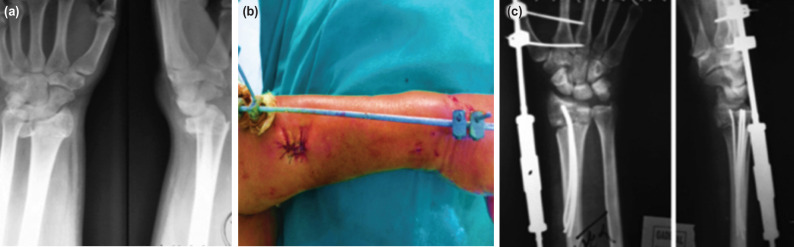
(a) AP and lateral radiographic images showing of a case with osteoporosis and comminuted distal radial fracture, (b) clinical photograph showing placement of an external fixator, (c) Radiographic views following final placement of IM wires and external fixator.

The need for external fixation was decided by the surgeon in cases in which the IM fixation with elastic wires alone would not suffice. While these fractures would be ideally suited for plate and screw fixation, the surgeon opted for this technique given the patients’ cost constraints. In all cases in which the fixation was done with a combination of IM elastic wires with external fixation, the fractures had presented with intra-articular extension of metaphyseal comminution and/or osteoporosis. It was determined that IM elastic wires with cast alone presented a risk of migration of the wires and/or fracture fragment(s) and potential risk of shortening. As such, the protocol at our centre is to use an external fixator as neutralising device in combination with IM elastic wire fixation in this specific group of patients that present with both clinical and socioeconomic challenges. In our series, 18 patients met these criteria and required the support of an external fixation device for at least four weeks to minimise subsidence and micromotion of the fractured bone segments (Fig. 3). A locally manufactured external fixator with 4mm rods and 2.5mm k-wires were used in our patients. Additionally, in eight patients with subluxated DRUJ, a percutaneous K-wire was inserted from ulna to radius after reduction of this joint. In three cases, the K-wire used for guiding reduction of the fragmented articular surface was retained for four to six weeks.

Post-operatively, a short-arm, well-padded, moulded plaster cast was applied in palmer flexion and ulnar deviation below elbow excluding metacarpophalangeal joints for at least four weeks in patients who did not receive an external fixation device. The cast allowed for unrestricted movements of the fingers and the elbow joint. All patients were evaluated for wound care and wire placement at 10 days following discharge and then at the four to six week follow-up when the cast/external fixator was removed. Following this, an elastic bandage was applied below the elbow joint for a period of two weeks for protection and stability in all patients. The patients were instructed to slowly begin wrist and forearm motions using a soft ball to clench it firmly as tolerated by each patient. Muscle strengthening exercises were initiated six weeks post-operatively. In four cases, the wires were removed at the patient’s request. This was done under local anaesthesia after an average duration of 18 weeks (range: 15 to 20 weeks) post-operatively.

In all cases, following radiographic evidence of callus formation or initiation thereof, the cast and external fixator were removed at four to six weeks in the clinic. All the patients were evaluated up to twelve months for pain, functional status, range of motion and grip strength of the affected wrists. Evaluation of the same parameters was also done on the patient’s contralateral healthy wrist for comparison. Radiographic evaluation was done during immediate post-operative period and at follow-up reviews to assess, radial inclination, ulnar variance and radial height. Both Sarmiento's modification of Lindstrom criteria and the demerit point system of Gartland and Werley were used for these evaluations^15,16^.

## Results

**Fig. 4:**
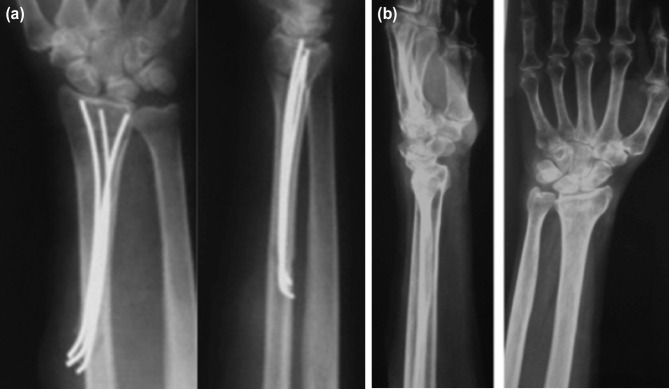
(a) Radiographic images showing union at the final follow-up with wires in-situ, and (b) following removal of wires.

**Fig. 5:**
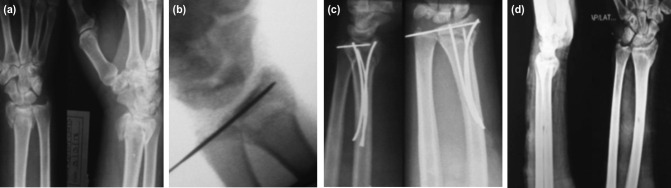
Radiographic images showing series of pre-operative to post-operative progression for one patient without the use of external fixation; (a) images showing comminuted distal radial fracture with instability of the DRUJ, (b) K-wire pinning across the DRUJ, (c) post-operative images following fixation, (d) and AP and lateral images at final follow-up showing radiographic union.

Fig. 4 and 5 are examples of pre and post-operative images of successful fracture union achieved in our study. Successful fracture union was observed in all 42 patients with average time to fracture union of 10.8 weeks (range: 8 to 14 weeks). There were no cases of delayed union (defined as at more than six months), or non-union in any of the patients. At the final follow-up, using the Sarmiento's modification of Lindstrom criteria, 12 patients (28.6%) had excellent, 23 (54.8%) good, 7 (16.6%) patients fair results, and none had a poor result (Table II). Using the demerit point system of Gartland and Werley. In comparison, 13 patients (31%) had excellent, 25 (59.5%) had good, four (9.5%) had fair results and none had a poor result. All patients reported comparable range of motion and grip strength between their operated and non-operated contralateral limb at the final follow-up.

**Table II:** Evaluation and results using Sarmiento's modification of Lindstrom criteria

Criteria Outcome	Residual Deformity	Loss of Radial Tilt (°)	Radial Shortening (mm)	Loss of Radial Deviation (°)	Number of Patients
Excellent	No deformity/insignificant	0	<3	5	12
Good	Slight	1-10	3-6	5-9	23
Fair	Moderate	11-14	7-11	10-14	7
Poor	Severe	At least 15	>12	>14	0

The total cost of treatment for each patient in our series was less than INR 1,000, including using stainless steel flexible wires, external fixator and casting; approximately INR 700 using IM wires with external fixation and approximately INR 500 for IM wires with casting.

One patient had uneventful IM migration of the wires, another reported a sensation of wire loosening, three others complained of joint stiffness and soft tissue irritation, and three reported relentless pain. These were considered as minor complications they resolved uneventfully by the final follow-up period. In our series, there was a loss of radial length of less than 3mm in 12 patients, 3-6mm in 23 patients, and 7-11mm in 7 patients. Despite such shortening, none of the patients reported adverse functional outcomes of their operated limbs.

## Discussion

Anatomical reduction and maintenance until union of fracture is the key to success in the treatment of distal radius fractures, and is usually a predecessor to determining the ability of patients to perform basic activities of daily living with absence or minimal pain in both the short- and long-term. There is no clear consensus on a standard treatment methodology for fractures of the distal radius as each case is very different from the other^17^. Non-surgical treatment options, such as a closed reduction with cast immobilisation should always be considered as the first line of treatment option in stable fractures presenting with minimal complications. However, if done incorrectly, or in patients with unstable or comminuted fractures, this technique may be more harmful than beneficial, eventually requiring a more drastic surgical procedure^18^.

There are many different techniques and implants available for surgical treatment of distal radial fractures. These include closed reduction and fixation with percutaneous pins, wires, semi-invasive IM nails and external fixators, or open reduction and internal fixation techniques that utilise a combination of bone plates and screws that can be placed either dorsally or volarly. Over the past few decades increasing number of surgeons are opting for the latter given the improved surgical outcomes that are reported in the literature, and also the fact that patients are increasingly becoming aware that they can regain a close to normal functionality with these newer implants^6,19^. However, this technique is not free of complications, and it can be highly cost-prohibitive, especially in rural areas in developing countries where access to quality healthcare is often limited to patients who are at the lower end of the financial spectrum^7^. In a prospective, randomised trial by McQueen *et al*, a total of 120 patients were divided into four equal groups of 30 patients each, who received one of four treatments: (i) closed re-reduction with a forearm cast for six weeks (non-surgical), (ii) open reduction with bone grafting and K-wire fixation with a forearm cast for six weeks, (iii) closed re-reduction with external fixation in which pins were inserted into the bone by an open technique, axis of wrist was locked for six weeks and the fixation was removed after six weeks, and (iv) same as (iii) but axis of the wrist was locked only for three weeks^20^. They observed that anatomical outcome measurements were best in the open reduction group and worst in the non-surgical group, however, and more importantly, there was no difference in the functional outcomes between any of the four groups. In another recent study by Saving *et al* comparing external fixation versus ORIF with a volar plating technique they observed no difference in patient functional outcomes at a follow-up period of three years^21^. They also noted that the rate of re-operation and occurrence of osteoarthritis was much higher in the ORIF group compared to the external fixation group. Therefore, utility of plate osteosynthesis needs additional studies demonstrating the cost-effectiveness and clinical outcomes in their use as described in recent literature. It may result in a more anatomic and radiographically aesthetic outcome but cannot be claimed as a superior procedure when compared to closed reduction and minimally invasive fracture fixation techniques as those too result in equivalent clinical outcomes.

Minimally invasive surgery continues to remain popular with both surgeons and patients, specifically as it relates to treating fractures of the distal radius. Not only is it cosmetically appealing to patients, but also to surgeons due to its technical and physiological benefits. Geissler *et al* advocate that both a minimally invasive percutaneous fixation with wires or limited open instrumentation-based fracture reduction guided by traction, ligamentotaxis and manipulation, preserve ligament and muscle attachments of the distal radius and carpal bones, maximise pain control, early rehabilitation and functional recovery while minimising morbidity, medical cost and lost work time^8^. In a majority of simple fracture cases of the distal radius, percutaneous k-wire pinning techniques such as trans-styloid fixation or Kapandji fixation with immobilisation are simple and cost-effective. However, patient outcomes with these techniques are at best reported to be varied and non-uniform, with a list of complications that include migration of pin in proximity to the radiocarpal or distal radio ulnar joints, skin irritation, pin tract infection, sensory nerve injury, tendon rupture, reflex sympathetic dystrophy and loss of reduction^22,23^.

In an attempt to minimise and possibly avoid these complications, and to adequately stabilise the fracture, Sato *et al* described a closed reduction technique with antegrade IM wire fixation in a series of patients with Colle’s type distal radial fractures^13^. They ensured that the distal ends of the wires were placed such that those would adequately maintain the fracture reduction and support the articular surface of the distal radius until healing occurred. Although the number of patients in their study was relatively small, they observed no complications related to tendon or nerve injuries, reflex dystrophy or pin loosening. The only two complications noted were skin irritation at the level of the forearm and protrusion of wire tips distally at the wrist joint. They did note that radial shortening in 48% of their patients was greater than 3mm and considered this technique to be ineffective in controlling that parameter adequately. Nonetheless, the major advantages of their technique included a low rate of occurrence of soft-tissue complications and prevention of dorsal angulation of fractures.

In a recent study, Mostafa adopted a slightly modified version of the technique proposed by Sato *et al* in a group of 28 patients presenting with AO/OTA Type A2-2, A3-2 and C1-2 fractures^13,14^. In his closed reduction and percutaneous antegrade IM fixation using wires, he ensured that the blunt ends of these wires were directed distally towards the subchondral bone beneath the radial articulating surface. The idea was that a blunt tip of a wire would not be able to penetrate the articular surface, which would eliminate the complication of the wire protruding through the joint, which is detrimental and can cause pain and other issues to the patient. Nonetheless, a wire breach into the wrist joint was observed in one patient in his study, with other minor complications such as skin irritation and a painful forearm bursitis. In his study, Mostafa observed excellent clinical outcomes in 61%, good in 25%, fair in 11% and poor in 4% of his patients^14^. The clinical outcomes and complications we report are comparable to his findings. Newer methods of IM nailing with new designs as reported in recent literature suggest that IM nailing can give comparable clinical results to current treatment modalities in extra-articular and simple intra-articular distal radius fractures. However, there is insufficient evidence to determine whether IM nailing with costly implants has any clinically important advantages compared to well-established alternatives^24,25^.

It is noted that fixation of closed, simple, single part, extra-articular Type A2 fractures should be attempted with a non-surgical approach first, with closed reduction and cast immobilisation. If that fails, the surgical techniques described herein should be explored for fixation. Our method is similar to that reported by Mostafa with the difference that we have added external fixation in some cases^14^. The distal blunt tips of the wires supported the articular surface of the distal radius by providing a three-point fixation aimed at minimising wire migration and penetration at the joint. The antegrade IM wires provide mechanical stability with support and fixation in the radial, middle and ulnar columns at the distal radius. In an attempt to further improve clinical outcomes and minimise complications in some of the more challenging cases, we made a modification to the technique wherein we augmented the antegrade IM wire fixation with an external fixation apparatus similar to those reported in the literature^26,27^. In our study, this was done in 18 out of the 42 patients who presented with osteoporotic and comminuted fractures of the distal radius. In these cases, IM fixation with wires alone did not suffice; therefore we used an external fixation apparatus to provide additional stabilisation, prevent radial shortening and minimise chances of intra-articular migration of the wires and/or bone fragments. An external fixator works by maintaining the reduction with distraction of ligaments that hold the fracture fragments in close approximation until healing, or a soft callus forms and stabilises the fracture. One may argue that these patients may be better suited for fixation with bone plates and screws. However, this is not always cost feasible for majority of the patients especially in rural areas. The cost of bone pates and screws at our centre range from INR 5,000 to INR 35,000, compared to a total cost of less than INR 1,000 for IM wires and external fixation combined. In our opinion, both methods provide the same results, and the latter is affordable for all patients. The average radial shortening in our series was 4mm, which could affect the function of articulation at the radio carpal and distal radio ulnar joints, but we observed that this shortening did not affect the functional outcomes in any of the patients in our study. Additionally, it is our belief that four to six weeks of immobilisation has no adverse effect on patient functional outcomes. This method can be used in a soft tissue compromised patient as it is based on indirect reduction and indirect stabilisation of fracture without disturbing the already compromised soft tissues, especially in high energy trauma cases.

While IM elastic wire fixation with casting is a standard technique, pin tract infection is a likelihood if adequate wound closure with proper hygiene is not maintained. As for the external fixation with IM elastic wires, these patients would ideally be suited for plate and screw fixation, but in our setting the cost would be prohibitive. This group of patients too is susceptible to increased risk of pin tract infection given the increased number of skin protruding rods/wires and presents with an overall increased level of patient discomfort until the device is removed.

The limitation of our study is that our patient follow-up duration is very short at one year. Longer term studies are needed to evaluate the benefit and risk profile of this treatment protocol. Another limitation is that the data we collected does not fit any statistical methods for us to analyse any trends or patterns. Although we observed residual joint stiffness in a few patients, with good physiotherapy all patient regained movements.

## Conclusion

Good to excellent functional and radiographic outcomes with easy to manage complications are achieved with the techniques described in this research study. The satisfactory results observed in this study are attributed to the early recognition of the type of injury and fracture pattern, careful patient selection, simplicity of the technique, careful post-operative management, and aggressive early rehabilitation. While open reduction with internal fixation would be ideal for a certain cohort of patients as described in this study, the use of elastic IM wires with external fixation is a viable alternative technique for socioeconomically challenged group of patients and can provide acceptable clinical outcomes.
